# Cacao agroforestry systems beyond the stigmas: Biotic and abiotic stress incidence impact

**DOI:** 10.3389/fpls.2022.921469

**Published:** 2022-07-28

**Authors:** Yeirme Y. Jaimes-Suárez, Albert S. Carvajal-Rivera, Donald A. Galvis-Neira, Fabricio E. L. Carvalho, Jairo Rojas-Molina

**Affiliations:** Centro de Investigación La Suiza, Corporación Colombiana de Investigación Agropecuaria—AGROSAVIA, Rionegro, Colombia

**Keywords:** AFS, black pod rot, frosty pod rot, light use efficiency, water use efficiency, witch’s broom disease

## Abstract

Low technological knowledge in production chains, global climate change, and misinformation are concrete threats to food security. In addition, these combined threats also trigger ecological instability in megadiverse areas of the world, especially in some cacao-producing countries in South America, where this crop plays an important socio-economic role, even being used to replace illicit crops. Accordingly, the use of agroforestry systems approaches has emerged as a good alternative to maintain productivity, add high-value commodities to producers, and provide important ecosystem services for sustainable agriculture. However, limitations associated with the competition for resources between the species composing the system, and the higher incidence of some diseases, have led many producers to abandon this strategy, opting for monoculture. In this review, we seek to gather the main information available in the literature, aiming to answer the question: what is the real scientific evidence that supports the benefits and harms of adopting agroforestry systems in cacao production? We seek to make critical scrutiny of the possible negative effects of certain associations of the agroforestry system with biotic and abiotic stress in cacao. Here, we review the possible competition for light and nutrients and discuss the main characteristics to be sought in cacao genotypes to optimize these inter-specific relationships. In addition, we review the research advances that show the behavior of the main cacao diseases (Witch’s broom disease, frosty pod rot, black pod rot) in models of agroforestry systems contrasted with monoculture, as well as the optimization of agronomic practices to reduce some of these stresses. This compendium, therefore, sheds light on a major gap in establishing truly sustainable agriculture, which has been treated much more from the perspective of negative stigma than from the real technological advantages that can be combined to the benefit of a balanced ecosystem with generating income for farmers.

## Introduction

Cacao (*Theobroma cacao L*.) is a native plant from the northern Amazon, currently cultivated in tropical regions of the world, especially in African countries (Motamayor et al., 2008). In America, the main producer countries are Ecuador, Brazil, Peru, and Colombia (FAOStat, 2021). Although this species comes from the undergrowth and has a wide variety of adaptations to grow under conditions of low light availability (Salazar et al., 2018), due to its phenotypic plasticity, some countries have found success in adopting monoculture cacao cultivation systems with full sun exposure (Lennon et al., 2021). Accordingly, cacao is grown in the world in both monoculture and agroforestry systems, and the two are considered to have different approaches to production and sustainability.

The cacao agroforestry systems (CAFS) can be classified as traditional or associated with planting arrangements. The traditional CAFS have intervened forests composed of different species in multi strata, where the cacao enters to replace one of the strata and the upper strata are kept as a shade. The CAFS with planting arrangements have a design with a homogeneous planting pattern involving cacao and one or more accompanying tree species to provide the shade. In these systems, the species selected for shading also represents a good part of the financial income of the plantation, as in the case of timber trees, which may be used to add high-value products to the system (Álvarez-Carrillo et al., 2012; Sambuichi et al., 2012). In the CAFS, the canopies of the forest trees may buffer environmental conditions, reducing air temperature, helping to retain moisture, and contributing to the improvement of physicochemical properties in the soil, as well as impacting the maintenance of biodiversity (Rojas-Molina et al., 2017; Marconi and Armengot, 2020). However, inadequate management of these canopies limits the entry of light and consequently limits photosynthesis, thus negatively affecting cacao production (Salazar et al., 2018).

The inadequate management of CAFS has generated a growing need to increase cacao productivity, and cacao monoculture has become an alternative to achieve this (Zuidema et al., 2005; de Almeida and Valle, 2010). However, this alternative also has disadvantages, such as the possibility of photooxidative stress, associated with excess light (Bassi and Dall’Osto, 2021), and the pressure for the use of water and fertilizers, which can also represent a negative factor for cacao sustainability if not properly managed. On the other hand, some authors positively correlate cacao diseases with CAFS (Andres et al., 2016). Although plant diseases can cause up to 100% yield losses in cacao, their severity depends on the level of management of the cacao plantation. The three main cacao diseases of particular interest are: (1) *Moniliophthora perniciosa*, the causal agent of the witch broom disease (WBD), (2) *Moniliophthora roreri*, which causes the frosty pod rot (FPR), and (3) *Phytophthor*a spp. which causes the black pod rot (BPR). Notwithstanding, environmental conditions are strongly linked to pathogenesis processes in agricultural systems (Cooke, 2006). There is still controversy over the use of shade trees in cacao crops and their contribution to the incidence of diseases, which is complex and requires a comprehensive understanding of the factors that favor their development. CAFS said to have microenvironments with high relative humidity and reduced light input, which favors the incidence of these diseases. This pre-conception has encouraged many producers to remove or reduce the number of shade trees from plantations (Marconi and Armengot, 2020). However, the life cycle of each of these pathogens differs from the others. In addition, the plant–pathogen interactions are non-linear and extremely complex systemic processes, which are highly affected by the environment, including the incidence of other ecosystemic factors such as the presence of natural biocontrollers.

Unfortunately, few studies compare CAFS and cacao monocultures regarding the advantages and disadvantages that may occur. This information would make it possible to resolve multiple aspects associated with the cost–benefit of cacao production, the socio-economic reality of cacao farmers, and the long-term sustainability of the production system. Hence there is a growing need to document these advantages and disadvantages, with agroforestry systems (AFS) being of special interest. In this current review, we explore how the AFS influences the physiology of cacao plants based on the use of light, water, and nutrients and highlight the possible correlations between cacao diseases and the agronomic model employed. Moreover, in this document, we attempted to scrutinize how forest tree management can favor or limit the development of cacao crop diseases. This information will contribute to the implementation of best practices and planting designs for the CAFS management, as well as help to select the best strategies for integrated management of cacao diseases.

## Cacao agroforestry systems vs. monoculture: Use of light, water, and nutrients

### Shading and light use efficiency in agroforestry systems

Light is one of the most crucial resources for the growth and development of plant, and consequently, this is a determining factor for crop productivity. Plants can absorb light in the photosystem complexes of thylakoids and employ this energy as the primary source for all assimilatory reactions, including nitrogen, sulfur, and carbon assimilation (Brestic et al., 2021). Consequently, these reactions are the primordial event for biomass formation that defines plant growth and productivity (Foyer et al., 2017; Lima Neto et al., 2021). Although all plants depend on light, the excess of this resource can be potentially harmful to plants. Accumulation of electrons in the thylakoid transport chain can promote the formation of reactive oxygen species that, can cause cell death, chlorosis, leaf abscission, and even plant death (Foyer, 2018). On the other hand, low light seriously limits the energy available for chemical reactions, crucial for plant growth and productivity (Lawlor and Tezara, 2009). Indeed, of the total light that falls on leaves, about 85% is absorbed by chlorophylls, and only about 5% of this energy is stored as organic matter (Pinnola and Bassi, 2018). Therefore, to survive in contrasting conditions of light fluctuation, as found in different agronomical designs, plants need to develop several molecular and biochemical strategies related to improving light use efficiency (LUE) in the shadow (AFS) or dissipate the excess of light to avoid oxidative stress (monoculture).

Cacao originated in understory regions of Amazon and is relatively adapted to tolerate shade conditions (González-Orozco et al., 2020). In shade conditions caused by the other plant leaves, as occurs in the understory, the taller plants absorb most of the light energy available, especially in the blue and red range. This absorbance contrasts with the light transmission of the longer wavelengths, with less available energy, mainly composed of the far-red range (Lorrain et al., 2008). The relative proportions between red and far-red are also important environmental signals capable of modulating processes in plants related to the best use of light and, consequently, signaling greater efficiency in the use of this resource (Puthiyaveetil et al., 2012; Goldschmidt-Clermont and Bassi, 2015). Among the strategies triggered by plants under these conditions are: (1) a relative increase in the proportion between antennas and reaction centers; (2) downregulation of dissipation processes such as non-photochemical quenching (NPQ); (3) anatomical changes related to leaf area and density; and (4) adjustments in the proportion between photosystems I and II (Coopman et al., 2010; Hughes et al., 2014; Ruban, 2015). Therefore, cacao plants need to exhibit one or more of these acclimatory characteristics to survive and produce in shade conditions.

**Figure 1:**
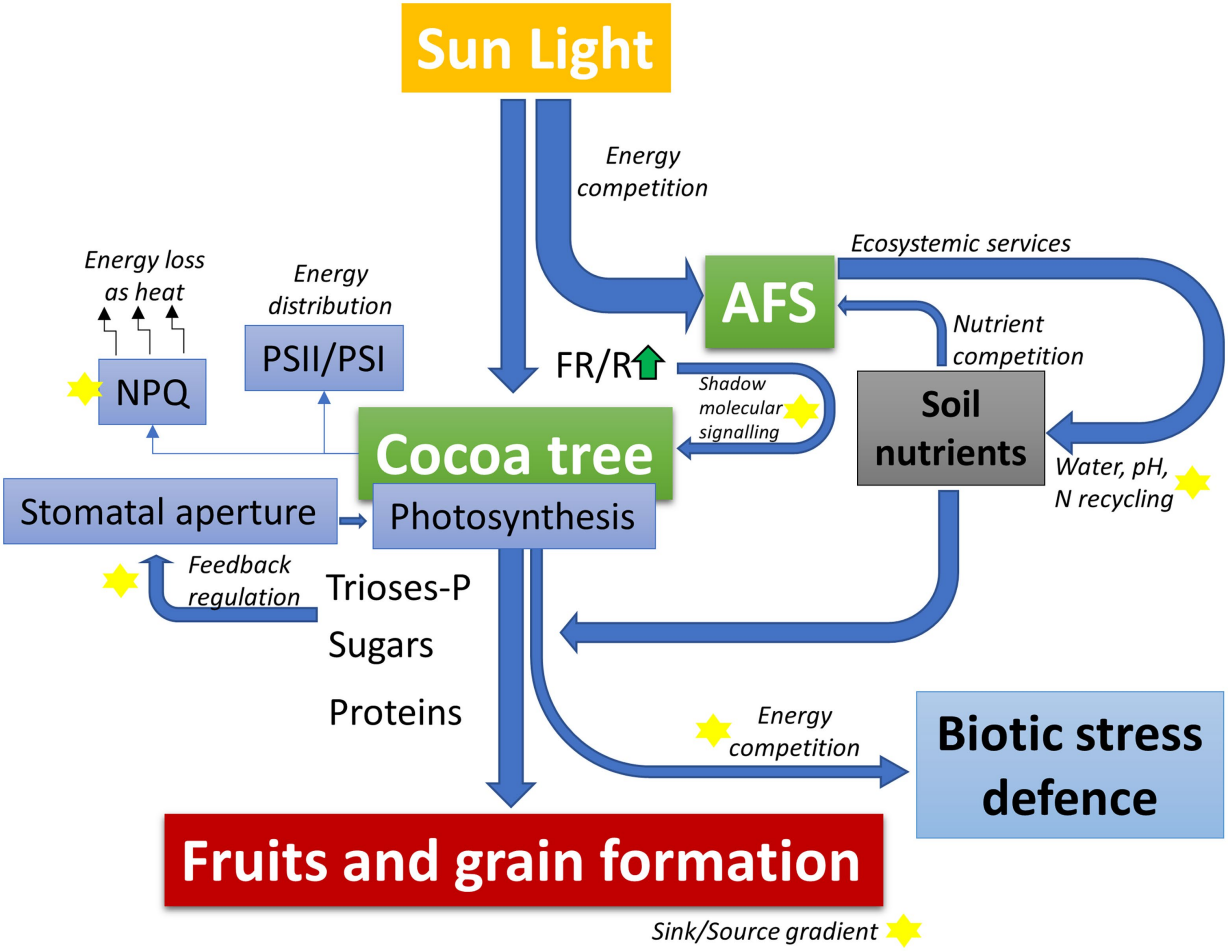
Schematic model highlighting the balance of light energy, carbon skeletons, and nutrients use distributed between activation of protection mechanisms against biotic stresses and productivity within the context of an agroforestry systems (AFS). In AFS, cocoa competes for light energy and soil nutrients with the shading tree species. This competition is directly counterbalanced by the ecosystem services provided by the shading trees that can favor water use efficiency and chemical soil properties (better availability of nutrients). Furthermore, differences in light quality (red/far-red light composition – FR/R) and contrasting balance in the levels of trioses-phosphate and other signaling molecules may activate physiological compensation pathways to optimize the energy balance of cocoa plants depending on the genetic background. These physiological compensatory responses may include adjustments in stomatal regulation, induction/relaxation of excess energy dissipation mechanisms (non-photochemical quenching, NPQ), and stoichiometric adjustments in the photosystem complexes (PSII/PSI). Finally, the resulting dynamic energetic and metabolic balance of the AFS-cacao model may directly compete with the energetic/metabolic demand of biotic stress defense mechanisms, thus determining the degree of limitation on the potential productivity of each genotype specifically. Yellow stars in the figure represent central processes for deeper investigation in AFS-cocoa models.

However, despite its adaptation to shaded environments, cacao also has remarkable phenotypic plasticity that allows it to grow and develop in conditions of full-sun exposition (Salazar et al., 2018; Baligar et al., 2021). This plasticity probably raises from the ability to modulate the expression of genes and regulate morpho-anatomic features that enable the plant to increase its photosynthesis capacity while promoting defense mechanisms against the excess of light, as expected for the full-sun conditions (Scheibe, 2019). Following the increased photosynthesis, cacao plants grown in full sun have higher productivity than plants grown in shaded environments (Mortimer et al., 2018). Nevertheless, studies have also shown that although productivity is increased under full-sun conditions, the productive life cycle is shortened by many years (Rajab et al., 2016). In addition, the incidence of some biotic stresses can become more frequent under full sun conditions than in shading, such as WBD. These responses in the life cycle and plant–disease interactions may also be possibly associated with increased competition in the energetic balance flow (Figure 1). Moreover, the ecosystemic services provided by AFS timber trees must be considered because of their environmental sustainability. Therefore, to maximize the long-term productivity of cacao, planting plots associated with AFS have been recommended and widely used in South America.

The average saturation light for cacao is about 400–500 μmol m^−2^ s^−1^ (Salazar et al., 2018). This intensity is relatively low, as it can reach 1,000 in beans and up to 1,500 μmol m^−2^ s^−1^ in some monocot crop species such as rice and wheat (Carvalho et al., 2014; Lobo et al., 2019). This data indicates that for most cacao varieties, luminous intensities above 500 μmol m^−2^ s^−1^ do not have positive effects on photosynthesis, otherwise may consist of excess energy. Considering that cacao cultivation may take place in tropical regions (Rodriguez-Medina et al., 2019), excess energy can indeed raise a problem for cacao farmers, therefore justifying its use in shaded AFS conditions.

The use of approximately 30–40% shading is strongly recommended for the proper management of cacao (Beer et al., 1998; Álvarez-Carrillo et al., 2012). However, light conditions below 400–500 μmol m^−2^ s^−1^ can potentially limit the availability of energy processed by the photosystem’s antennas, demanding a greater LUE to ensure growth and productivity. In addition, other factors such as the altitude, cartesian orientation, and the frequent incidence of clouds in the region must be considered to maximize light availability in AFS (Salazar et al., 2018). Therefore, cacao plants presenting higher LUE may be more productive under shader AFS conditions. In this way, the arrangement of the CAFS is of fundamental importance to ensure that the appropriate luminous intensity will be available for cacao growth and productivity (Mortimer et al., 2018). Exploiting the great phenotypic plasticity and genetic diversity presented by cacao plants may also allow further selection of genotypes with higher LUE. This approach could lead to a new, more dynamic, and efficient CAFS design.

The LUE, however, cannot be taken as a static process since it is extremely dynamic. For example, recent studies in coffee AFS have reported that even when irradiance was reduced by 60%, coffee light-use efficiency was increased by 50%, leaving net primary productivity stable across all shade levels (Charbonnier et al., 2017). In these plants, the endogenous features and other environmental conditions have more effect on productivity than the light intensity *per sea*. The authors concluded that the age of plants and the interspecific competition for water and soil nutrients among coffee and the tree species were more relevant for crop productivity (Charbonnier et al., 2017). In cacao, there are no studies regarding the LUE plasticity as a response to different microclimate conditions and interspecific interactions inside the different CAFS plots. However, some studies approached photosynthetic responses under contrasting CAFS designs (Gómez-Yarce et al., 2020) and evaluated contrasting cacao varieties (Agudelo-Castañeda et al., 2018). These studies also reinforce the great plasticity of cacao genotypes and the importance of interspecific interactions in CAFS designs.

The mechanisms underlying the LUE adjustment in plants as a response to different light intensities are still not completely elucidated to date. Source–sink relationships, which are strongly associated with carbon allocation during the reproductive phase, could represent a decisive factor in determining the LUE in shaded plant species (Cannell, 1971). According to this hypothesis, the light use efficiency is adjusted in response to the activation of sink-source gradients. In this case, the energy demand, mainly related to fruit production, would be ultimately the limiting factor for determining the LUE. This response, in turn, would be highly determined by other environmental conditions, besides light, which in fact would be the driving force behind the regulation of the LUE (Charbonnier et al., 2017). Many molecular mechanisms are known to directly compete for the energetic flow driven to the sugar biosynthesis, consequently affecting indirectly the allocation of carbon to fruits (Figure 1). For example, the non-photochemical quenching (Murchie and Ruban, 2020) and the photorespiratory activity (Guilherme et al., 2019) may drastically affect the energy balance in the leaves. Studies aiming to understand the physiological, molecular, and genetic components associated with these processes in cacao, especially within an integrative and multidisciplinary context associating the possible interactions of pathogens within the CAFS competitive model are still needed.

### Cacao agroforestry systems ecosystemic services: Water and nutrient availability

In the CAFS, the interspecific competition for water and nutrients also must be considered (Niether et al., 2019). Different species of timber trees are capable of interfering in many ways with the fertility and microbiology of soils, consequently affecting the availability of nutritional resources for cacao to complete its biological cycle of growth and development. However, several advantages of CAFS have been documented. These can decrease air and soil temperatures compared to monoculture, reducing evapotranspiration (Mortimer et al., 2018). Thus, CAFS can play ecosystemic services that contribute to increasing the resilience of cacao plants to climate variability (Green et al., 2021).

**Figure 2:**
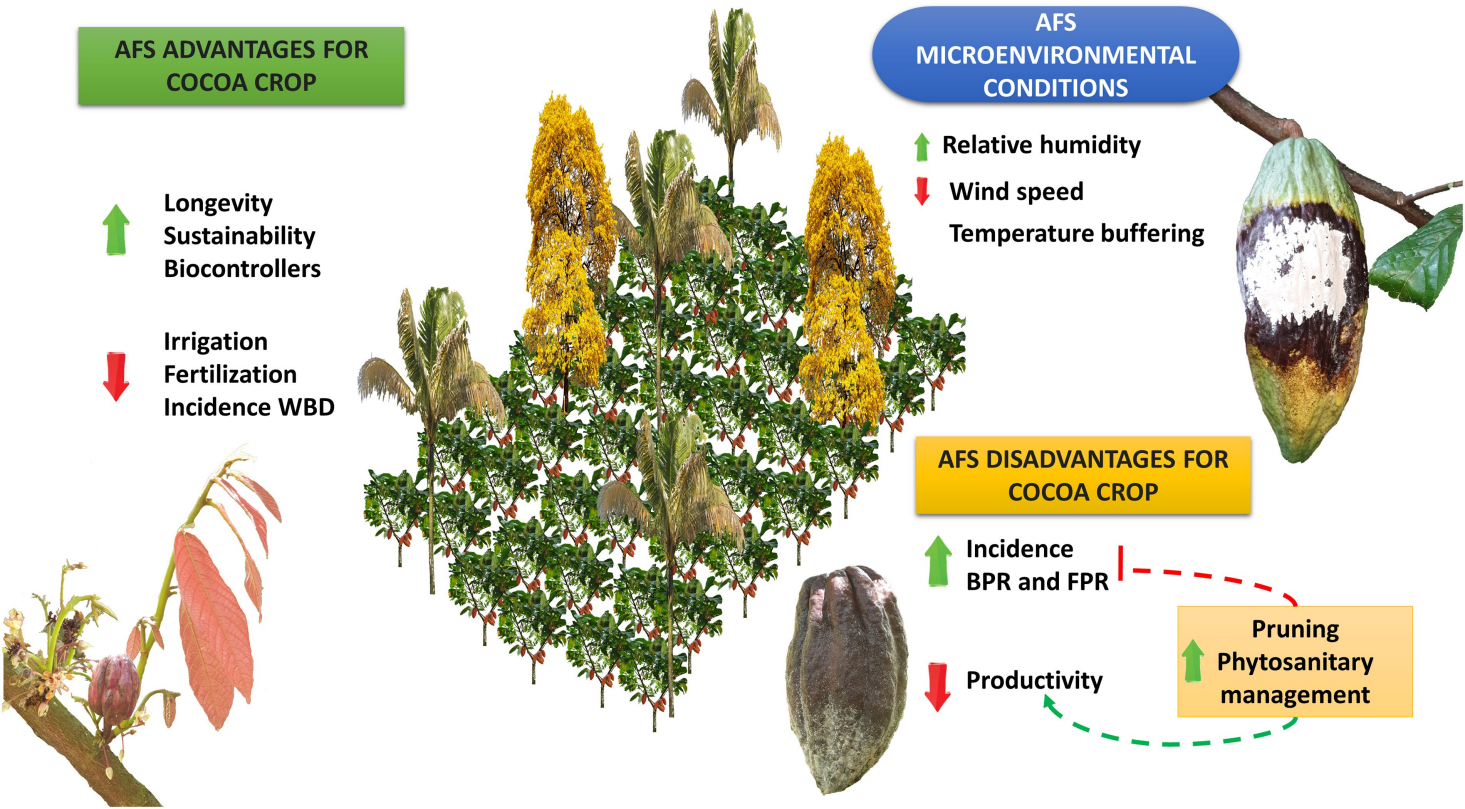
Impact on the development of diseases and cacao productivity in agroforestry systems as compared to monoculture. Green arrows mean a favored process and red arrows mean an unfavorable process. AFS promotes specific changes in the cocoa microenvironment, especially related to higher humidity and potentially a greater buffering of temperature changes, despite it may reduce the wind speed inside the system. These changes have the potential to induce an increase in the longevity of cocoa plants, better use of pest and disease biocontrollers, and ultimately promote environmental sustainability. In addition, it may promote a lower need for irrigation and fertilization, as well as a lower incidence of witch’s broom disease (WBD), impacting positively the producers’ costs. However, in parallel AFS models have a great potential to negatively impact cocoa productivity due to higher susceptibility to black pod rot (BPR) and frosty pod rot (FPR) if pruning and phytosanitary management are not carried out properly.

Likewise, different tree species have distinct requirements for water resources, which can be an extremely important factor in conditions in which this resource becomes scarce. In a CAFS, cacao productivity will be determined by the intersection of four main factors: (1) the specific genotype genetic background of cacao, which determines the degree of plasticity of the phenotypic response under environmental oscillations; (2) the specificity of the habit of the accompanying trees; (3) the biophysical characteristics of the environment where the system is developing, including temperature, altitude, and rainfall; and (4) the possibility of the occurrence or not of biotic stress; which are all subject to human factor interference (Figure 2).

According to the AFS hypothesis, timber trees can interfere (allelopathy + competition) with the crop and generate complementary effects (Hierro and Callaway, 2021). Through allelopathy and competition, timber trees can deplete some nutrients or release potentially phytotoxic substances, with negative effects on the growth and productivity of some crops (Scavo et al., 2018). On the other hand, favorable interspecific interactions can also make some mineral resources more available by altering the chemical properties of soils, for example (Hosseini Bai et al., 2017). It has even been reported that some AFS models can affect the pH and increase the availability of organic carbon, inducing the microbiological quality of soils and thus favoring the associated crops (Bai et al., 2016). Likewise, the AFS can contribute to several processes to improve the water use efficiency of AFS crops, reducing the need for irrigation complementation (Hatfield and Dold, 2019). Therefore, the potential ecosystem services related to better use of water resources performed by the AFS model should be considered as an extremely positive factor in the balance that includes disease incidence, management cost, and productivity, when compared with the monoculture system, especially under the projected scenario of global climate changing.

In cacao, Zuidema et al. (2005) found that the annual radiation and the accumulated precipitation of the dry seasons explain 70% of the variation in production concerning the simulated potential, in more than 30 localities around the tropics, with a strong correlation between the precipitation of the driest months and the annual dry cacao beans yield. These data confirm the importance of water availability for cacao productivity. However, despite the high importance of the water resource in cacao, there are few publications of practical value on the responses of the crop to drought or irrigation (Jegadeeswari and Kumar, 2019; Hebbar et al., 2020), especially under agroforestry arrangements. Niether et al. (2019) reported that the AFS modifies the hydric relations of cacao through changes in the percentage of transmitted light, effective precipitation, and microclimate, where evapotranspiration reaches values of 5.12 mm day^−1^ in the monoculture and 4.51 mm day^−1^ in an AFS. Experimental evidence has shown that fully exposed cacao trees have shorter production cycles, leading to progressive plant deterioration, loss of vigor (proliferation of some diseases and insect attacks), and finally death (Alpízar et al., 1986).

In addition, at full exposure, factors such as high radiation and air temperature can have negative effects on cacao plants (Salazar et al., 2018). When the cacao leaves are exposed directly to the sun, there is a 40% increase in the temperature of the leaf, about the leaves under shady (28°C). This results in a high increase in photorespiration from leaves exposed to full sun (Huang et al., 2016), increasing the possibility of water stress. In addition, if the water requirements are not met, the imbalance between the contribution of incident light energy in the leaves and the consumption by metabolic processes can generate photoinhibition, photodamage, and irreversible effects on plant growth and development (Cunha et al., 2019). This problem is of particular importance for Colombia, which can present variations of more than 1,000% of daily light intensity (Salazar et al., 2018), reaching peaks of 2,500 μE m^−2^ s^−1^ in some regions. In fact, in stressed cacao plants, at least a 25% reduction in CO_2_ assimilation rates may occur, while transpiration rates can decrease by 40% due to stomatal closure (Agudelo-Castañeda et al., 2018), which can be aggravated by the occurrence of parallel biotic stress and nutritional deficiencies. Moreover, CAFS are completely dynamic systems. The time and intensity of pruning are essential to balance light and water availability in environmental conditions that varies seasonally to conserve microenvironments for cacao production with less exposure to unfavorable climates (Niether et al., 2019).

Therefore, despite possible productivity reductions, which may be also compensated by more appropriate management procedures, AFS can benefit the long-term durability and stability of the associated crops. In addition, AFS strategies can bring cost-saving benefits in the use of fertilizers and irrigation (Wartenberg et al., 2020). Nevertheless, the possible correlation between the use of AFS and the incidence of biotic stress would still be by far the most controversial point for its widespread use in cacao production systems. In the next sections, this review will scrutinize facts and myths about the disease susceptibility of AFS associated with cacao.

## Cacao agroforestry systems vs. monoculture: Development of cacao diseases

### Witch’s broom disease

The WBD, caused by the fungus *M. perniciosa*, may generate losses of up to 100% of production, even causing the abandonment of the crop (Pereira et al., 1996; Meinhardt et al., 2008; Andres et al., 2016; Sousa Filho et al., 2021). Four biotypes (C, H, L, and S) are reported for *M. perniciosa*, which are classified by their reproductive biology and host specificity. Biotype C infects plants of the Malvaceae family, including the genus *Theobroma* (*T. cacao*, *T. grandiflorum*, *T. bicolor*, *T. obovatum*, *T. microcarpum*, *T. speciosum*, *T. subincana*, *T. sylvestris*; Meinhardt et al., 2008). Two pathotypes have been reported within biotype C identified as pathotypes A and B, where pathotype A is the most virulent and is found distributed in Colombia, Bolivia, and Ecuador. On the other hand, pathotype B is found in Brazil and Venezuela and presents less virulence (Gramacho et al., 2016).

*Moniliophthora perniciosa* is a hemibiotrophic pathogen, characterized by having two different phases in its life cycle (Evans, 2016). Remarkably, the pathogen is an obligate parasite whose mycelium is not infective and only spores are capable of inducing infection (Kilaru and Hasenstein, 2005). After infection, *M. perniciosa* colonizes the host as a biotrophic pathogen, affecting new shoots, flower cushions, and fruits, presenting various symptoms. Vegetative brooms develop on the stem and branches, where the axillary and lateral buds suffer hyperplasia, producing elongated and swollen stems with flaccid leaves and long petioles (Silva et al., 2002). After 3–4 months, the leaves fall off, leaving only the withered branches that resemble a broom. In the flower buds, multiple parthenocarpic flowers and fruits can form, shaped like carrots or custard apples that quickly necrotize, becoming woody, hard, and “mummified.” In developing fruits, irregular dark brown lesions are formed that, unlike *M. roreri*, harden and remain free of mycelium (Aime and Phillips-Mora, 2005; Evans, 2016). Finally, the pathogen begins its necrotrophic or saprophytic phase in the infected dead tissues, producing the fungus fruiting bodies, called basidiocarps. If favorable environmental conditions are present, the pathogen releases the basidiospores, which are dispersed with the help of the wind to start a new cycle of infection (Evans, 2016).

Some studies report on the incidence of WBD in cacao plantations grown on CAFS, coinciding that agroforestry plantations with cacao have a lower incidence of WBD (Table 1; Evans, 1998). Consequently, it is possible to assume that as cacao crops gradually become monocultures, the incidence of WBD may increase significantly (Jacobi et al., 2013). When cacao plantations are exposed to the full sun without shade trees, this leads to an increase in bud and flower formation and, associated with this, an increase in susceptibility of meristematic tissues (Milz, 2006). The ideal conditions for this pathogen to progress in monocultures are based on the change in the environment of the pathosystem, which also implies a low activity of biocontrollers (Figure 2). In this sense, CAFS harbor native plant species that contribute to the formation of multiple antagonistic microorganisms and saprophytes, which have evolved together with *M. perniciosa*. Generally, these microorganisms grow under the canopy of the trees, in the phylloplane, and in the soil (organic matter), which under CAFS environmental conditions are protected from direct sunlight, temperature, and desiccation (Schroth et al., 2000; Vaast and Somarriba, 2014; Armengot et al., 2020). At the same time, intensive technical management of crops at full exposure promotes overproduction of flowers and branches, in turn increasing potential sites of infection. This situation, combined with the adverse factor caused by overexposure to light and the increase in wind speed, may facilitate the conditions for the development of *M. perniciosa* infection (Evans, 1998; Schroth et al., 2000). Relative humidity also plays an important role in the formation of brooms. If this climatic variable is stable and does not suffer strong alterations, it has been shown that the occurrence of brooms is favored (Nunes et al., 2002).

**Table 1:** Literature review on the use of agroforestry systems (AFS) in cocoa production, evidencing its effects on the incidence of diseases and potential impact on productivity.

Pathogen	Impact on disease development	AFS impact on cocoa crop productivity	Country	Reference
*Phythpthora spp.*	There are no significant differences compared to monocultures if cultural practices are applied opportunely	Positive-conditioned	Bolivia	Armengot et al. (2020)
*Phythpthora spp.*	There are no significant differences compared to monocultures if cultural practices are applied opportunely	Positive-conditioned	Ghana	Leitão (2020)
*Phythpthora spp.*	The incidence and severity of black pod disease increases proportionally with increasing shade level.	Negative	Cameron	Ambang et al. (2019)
*Phythpthora megakarya*	Companion species of AFS are hosts and spread the disease.	Negative	Cameron	Holmes et al. (2003)
*Phythpthora megakarya*	Companion species of AFS are hosts and spread the disease.	Negative	West Africa	Opoku et al. (2002)
*Phythpthora palmivora, Phythpthora megakarya*	Companion species of AFS are hosts and spread the disease.	Negative	Ghana	Akrofi (2015)
*Moniliophthora roreri*	There are no significant differences compared to monocultures if cultural practices are applied opportunely	Positive-conditioned	México	Torres de la Cruz et al. (2011)
*Moniliophthora roreri*	There are no significant differences compared to monocultures if cultural practices are applied opportunely	Positive-conditioned	Perú	Krauss and Soberanis (2001)
*Moniliophthora roreri*	There are no significant differences compared to monocultures if cultural practices are applied opportunely	Positive-conditioned	Bolivia	Armengot et al. (2020)
*Moniliophthora perniciosa*	The incidence and severity of witch’s broom disease decrease proportionally with increasing shade level.	Positive	Ecuador	Evans (1998)
*Moniliophthora perniciosa*	The incidence and severity of witch’s broom disease decrease proportionally with increasing shade level.	Positive	Perú	Krauss and Soberanis (2001)
*Moniliophthora perniciosa*	The incidence and severity of witch’s broom disease decrease proportionally with increasing shade level.	Positive	Bolivia	Jacobi et al. (2013)
*Moniliophthora perniciosa*	The incidence and severity of witch’s broom disease decrease proportionally with increasing shade level.	Positive	Venezuela	Hernández-Villegas (2016)
*Moniliophthora perniciosa*	The incidence and severity of witch’s broom disease decrease proportionally with increasing shade level.	Positive	Bolivia	Andres et al. (2016)
*Moniliophthora perniciosa*	The incidence and severity of witch’s broom disease decrease proportionally with increasing shade level.	Positive	Bolivia	Armengot et al. (2020)

A positive impact means that there was no significant difference concerning the incidence of a given disease, while a negative impact indicates that there is a higher incidence of the disease, in both cases comparing the AFS model with the monoculture. A positive-conditioned impact means that there are no differences in the disease progression comparing AFS and monoculture systems if adequate cultural practices are opportunely adopted.

The higher constant humidity allows the basidiospores to germinate and penetrate the germ tube. Studies have reported that at least 4–6 h of high humidity (100% RH) is required for the germination of basidiospores, but after 14 h of exposure to 100% RH, the successful infection reaches the maximum percentage (Frias et al., 1995). This condition is favored in the cultivation of cacao under shade or in seasons of high rainfall. At the end of the necrotrophic phase, brooms require a particular condition to generate their basidiocarps, which requires alternation between wet and dry periods to stimulate the generation of basidiocarps and develop the infection. The environmental conditions related to monoculture promote rapid drying of the brooms, increasing the sporulation period and inoculum potential (Schroth et al., 2000; Meinhardt et al., 2008; Evans, 2016). In this sense, CAFS is presented as a viable alternative for mitigating the economic effects produced by the WBD. The CAFS promotes adverse environmental conditions for the spores to spread and the development of the disease, damping the temperature and ventilation on the cacao plantations (Milz, 2006; Niether et al., 2020).

### Frosty pod rot

The FPR or moniliasis, caused by the hemibiotrophic pathogen *Moniliophthora roreri*, is classified as the most important cacao disease in America and the Caribbean. This disease is considered more destructive and difficult to control than the BPR and the WBD and may cause up to 90% of economic losses, leading to the abandonment of important grain-producing regions such as Ecuador, Peru, Colombia, and Costa Rica (Krauss and Soberanis, 2001; Phillips-Mora and Wilkinson, 2007; Bailey et al., 2018). Its high dispersal capacity (more than seven billion spores in a diseased fruit) and its ability to adapt to a wide range of environmental conditions (Phillips-Mora and Wilkinson, 2007), make it an aggressive pathogen that has had a fast distribution over the south and central America. After penetration, the spores generate oily spots, hyperplasia, manifesting as swellings (humps), premature ripening of the fruits, and brown spots, after 4–5 days on the spot a layer of white mycelium develops that becomes darker as spores mature. Finally, the fruits dry and mummify, remaining attached to the tree (Jaimes and Aranzazu, 2010; Bailey et al., 2018).

The response of moniliasis to the shading conditions provided by the CAFS is positive for the disease, finding that the incidence of the disease grows proportionally to the increase in the level of shade. The shady environment provided by the AFS implies damping of temperature, a reduction in the availability of light and wind speed, and consequently an increase in relative humidity (Niether et al., 2020). These microclimatic conditions provided by the CAFS positively respect the intensity and development of the disease, since the FPR is favored by an increase in relative humidity (> 90%) and by lower temperatures (20–26, 9°C; Torres de la Cruz et al., 2011). In Mexico, it is reported that a 50% reduction in the shade, independent of the management used, can lower the incidence of FPR by up to 20%, and it is also found that if integrated management is applied, the incidence could be reduced by up to 68% (Torres de la Cruz et al., 2011). Similarly, in Peru, it was found that the incidence of FPR is 25% higher in the treatment with dense shade than with crops with full sun exposure (Krauss and Soberanis, 2001). In Bolivia, they reveal that the successional agroforestry system developed a higher incidence of FPR than monoculture (Armengot et al., 2020). However, both studies also reveal no significant differences in the incidence of FPR when integrated disease management is applied, regardless of the shade level of the crop (Table 1).

On the other hand, the organization and selection of the plant community that integrates the CAFS is an important criterion in the development of the disease, where there are species that generate a drier microclimate that favors the dispersal of spores, such as the case of *Erythrina poeppigiana*, which carries a greater quantity of spores in the air concerning *Inga edulis* and *Gliricidia sepium*. This condition is probably due to the physiological properties of the species since *E. poeppigiana* supports a greater intervention of the tree through pruning (Meléndez and Somarriba, 1999). Nevertheless, this companion species should not be disqualified, since by generating drier conditions, infection with *M. roreri* is also unfavorable. It can be said that the incidence and severity of the FPR also depend on the cacao genotype, also referred to as “clone” (Jaimes et al., 2019), and the diversity and aggressiveness of the *M. roreri* isolate (Jaimes et al., 2016), which will determine the level of intervention required for the cacao plants. Also, it has been found that the shade tree density and cacao tree density have a negative correlation with the intensity of the FPR. To establish CAFS that are not conducive to FPR, the architecture of the shade tree should be considered and its distribution should be moderate and uniform within the plantation (Gidoin et al., 2014). Therefore, the CAFS and monoculture strategies present both positive and negative characteristics related to the progression of the disease, its success will depend on its management, the cacao genotype, and the planting design. Low or high incidence of FPR may also depend on the microclimate conditions generated since the pathogen requires different climatic conditions (humidity and temperature) to complete each phase of its cycle (Evans, 1981).

### Black pod rot

Black pod rot caused by *Phytophthora* spp. is the disease with the greatest economic importance in the cultivation of cacao in the world, causing losses of 30% of the production, in addition to causing the mortality of up to 10% of the trees annually. Producing cankers on the stems (Guest, 2007). So far, seven species of *Phytophthora* have been reported related to the etiology of BPR disease in cacao cultivation, being *P. palmivora* the cosmopolitan species of the group (Akrofi, 2015), with pantropical distribution (Perrine-walker, 2020). The other species have only been found in specific countries or geographic regions such as South and Central America: *P. capsici, P. citropthora,* and *P. tropicalis* (Barreto et al., 2015; de Bahia et al., 2015; Fernández Maura et al., 2018), Brazil: *P. theobromicola* (Decloquement et al., 2021), Venezuela: *P. megasperma* (Molina et al., 2016), West Africa: *P. megakarya,* and *P. katsurae* (Guest, 2007; Liyanage and Wheeler, 2007). Among these species, *P. megakarya* is the most important economically in the world, since it is reported in the African countries with the highest cacao production (FAOStat, 2021). The disease may cause losses of 60–100% of production in countries such as Cameroon, Gabon, and Ghana (Deberdt et al., 2008; Akrofi et al., 2015; COCOBOD, 2019). In turn, *P. megakarya* produces significant effects on production costs concerning other species since requires the application of fungicides (Opoku et al., 2002), costing up to 21 million fungicides packages per year in Ghana (COCOBOD, 2019).

Phytophthora infection in nursery seedlings causes leaf necrosis and root rot, while stems and branch infections in the field cause cankers. Every stage of fruit development, from flowering to ripening, is susceptible to infection and produces necrosis, but immature fruits are the most susceptible (Akrofi, 2015). The first symptoms appear 2–3 days after the infection, consisting of a translucent chlorotic spot, then the spot turns brown, and spread rapidly to cover the entire fruit after 10–14 days (Luz and Silva, 2001). Infected fruits in an advanced stage develop a characteristic fishy odor 3–5 days after the appearance of the first symptom, which is associated with the mycelium spot with whitish spores (Barreto et al., 2015).

Black pod rot is positively influenced by humid and cold conditions (Akrofi, 2015), especially when the temperature is below 20°C and the relative humidity is greater than 85% (De Oliveira and Luz, 2005). These are conditions that can occur under CAFS, and based on this data, we could assume a negative influence of CAFS on the control of this disease. It has been reported that CAFS, which include fruit trees, palms, and forests, have a microclimate that differs from the monocultures conditions with a full solar exposure (Niether et al., 2020). Most farmers relate the microclimatic conditions generated in CAFS as one of the causes of a higher incidence of fungal diseases, compared to monocultures with full sun exposure (Armengot et al., 2020).

Diverse theories have been reported on the influence of CAFS in the development of *Phytophthora* spp. in cacao plants, which include investigations on the proportion of shade provided by various AFS and its impact on the development of the BPR disease (Table 1). These studies reveal the influence that some companion species of AFS may have on the dissemination and inoculum potential of *Phytophthora* spp. in cacao cultivation, some of these species may even work as hosts of the pathogen. Indeed, from the root of nine species often used in the CAFS in Ghana was isolated *P. megakarya* (Akrofi et al., 2015). These species are reported in the floristic composition of AFS with cacao from Africa (Sonwa et al., 2007) and are widely used in agroforestry systems in American countries, such as *Musa paradisiaca, Persea americana, Mangifera indica* and *Carica papaya* (Ramírez-Meneses et al., 2013; Guiracocha et al., 2016; Peña et al., 2019; Mercedes Ordoñez and Rangel-Ch, 2020; Jaimes et al., 2021). Other species frequently used in this system in Africa are also reported as hosts of *P. megakarya* and *P. palmivora*, among which we can find forest and palm species such as *Irvingia gabonensis*, *Funtumia elastica*, *Sterculia tragacanta, Ricinodendron heudelotii,* and *Elaeis guinnensis*; shrubs and short plants such as *Dracaena mannii, Xanthosoma saggitifolium, Colocasia esculenta, Athyrium nipponicum* and *Ananas comosus* (Opoku et al., 2002; Holmes et al., 2003; Akrofi et al., 2015). These reports suggest that the influence of the inoculum potential of *Phytophthora* spp., which is harbored by the companion species of cacao, could explain the inefficiency in the control of the disease (Akrofi et al., 2015). In addition, to adjust disease management strategies, it is necessary to propose control methods that not only contemplate the management of the disease in cacao but also in the accompanying species, especially in those that can be a primary inoculum reservoir since these plants are asymptomatic.

On the other hand, there is little information on the levels of shade that favor the FPR disease. However, in one of these studies, it was found that a diverse floristic composition in CAFS can cause problems with spatial structure when its distribution in the plantation is heterogeneous. The heterogeneous distribution of trees can project dense shade in most cases, and thus generates predisposing microclimate conditions for the development of pathogens (Figure 2). With this, it can be concluded that the incidence and severity of *Phytophthora* spp. in CAFS increase proportionally to the shade level (Ambang et al., 2019). Additionally, the incidence of the FPR is not always related to high shade density, in crops with few but diseased trees the incidence of this disease is increased as well as the number of diseased mature pods (Armengot et al., 2020; Leitão, 2020). Not always, the development of FPR is related to the high level of shade, it is also necessary to consider an adequate distribution of shade trees to regulate the level of shade in the plantation. Inadequate distribution can generate microclimates with high relative humidity and low wind speed (Niether et al., 2020), predisposing conditions to the FPR development. Nonetheless, some authors report that there is no difference between disease incidence in PBS and incidence in a monoculture, as long as proper management practices are implemented on the plantation (Armengot et al., 2020; Leitão, 2020).

## Biotic and abiotic stress interactions in cacao

In their natural environment, plant exposure to combined biotic and abiotic stress conditions is a rule rather than an exception. Indeed, this is a hot topic in literature (Suzuki et al., 2014; Kissoudis et al., 2016; Fichman and Mittler, 2020) and the complex responses triggered by these combined events are still very poorly understood. Interestingly, the combination of conditions potentially causing stress in plants does not promote solely additive responses, that is, the sum of two events that alone have the potential to induce stress in plants does not necessarily generate a more severe stress response. Yes, in fact, on many occasions additive responses have been observed, for example in cowpea, where pre-exposure to saline stress can generate susceptibility to diseases caused by viruses in known resistant genotypes (Varela et al., 2019).

However, the combination of stress has also been shown to be interactive, that is, when changing a specific potentially stressful environmental condition, systemic plasticity responses at different levels in plant agents can trigger mechanisms such as assays that promote a pre-acclimatization of the organism to others. Types of future stressful conditions. This phenomenon is known as plant stress memory (Nikiforou and Manetas, 2017; Pintó-Marijuan et al., 2017; Auler et al., 2021) and is directly related to the cross-tolerance processes that have been observed in plants (Carvalho and Silveira, 2020). For example, recently Chávez-Arias et al. (2021) highlighted that the combination of some abiotic stresses and arthropod herbivory in maize may increase the production of volatile and non-volatile compounds resulting in improved response to pest infestations.

In cocoa, however, in-depth studies on the combination of biotic and abiotic stresses are still rare. On the one hand, yes, we have very instigating studies as reported in the present review (Beer et al., 1998; Schroth et al., 2000; Niether et al., 2019, 2020) which compare, for example, the influence of the environment associated with CAFS with that of the environment associated with monoculture in terms of incidence or not of the typical cacao tree diseases. These approaches allow us to discern with a high degree of clarity that yes, in some situations, especially when associated with inadequate management of CAFS, it is possible to observe a higher incidence of diseases in cacao plants. However, when comparing the CAFS environment with the monoculture environment, several abiotic factors can vary simultaneously, such as light, humidity, temperature, and wind intensity, thus making it impossible to distinguish the interactive factors between each of the components of the environmental system. Therefore, this information still presents a very important gap in the understanding of the effects of combined stresses in cacao and deserves future investigation.

We are currently facing a global crisis associated with the imminence of the irreversible effects of global climate change, which should be further exacerbated by the collateral consequences triggered by the global pandemic of COVID-19. In this context, more drastic climatic events such as rains and dry seasons (Lahive et al., 2019), increase in fertilizer costs, and high prices of agricultural products stand out, threatening global food security (Roubík et al., 2022). Therefore, understanding the processes of interaction between combined biotic and abiotic stresses in species where these processes are still poorly understood, such as cocoa, may anticipate early strategic actions that may be of great importance.

## Economic return: CAFS vs. monoculture

In terms of economic return, there is a negative perception of CAFS vs. monocultures. This is because the comparison is made based only on cocoa bean production in the short term. Clearly, in this period there is a higher production in monoculture. However, in the long term, cocoa production under agroforestry trees shade will be compensated because the productive life of a tree is extended. This is because the leaves of the cocoa tree under shade are longer-lived since their exposure to solar radiation is reduced (Niether et al., 2020).

On the other hand, to maintain adequate productivity of monocultures, chemical fertilization is required, since with organic fertilization there are no differences with the production of a CAFS. In the CAFS, there are no differences in production between the use of organic and inorganic fertilizers. This can be associated to the fact that fertilization does not have the same relevant effect as the availability of light in agroforestry systems. Therefore, it is believed that to improve cocoa productivity in agroforestry systems, shade tolerant varieties should be selected, as well as adequate conditions to increase the size of the pollinator population (Armengot et al., 2016).

Economic point of view, agroforestry is less profitable than monoculture, but the economic benefit is given in the longer term and at the level of society in general by establishing agroforestry systems with an adequate level of shade, since the environmental services offered are greater and sustainable in the long term (Owusu et al., 2021). In addition, agroforestry systems provide additional income from shade trees (timber, fruit, medicinal, etc.) and reduce the use of external inputs (herbicides and large amounts of fertilizers) and, for many small producers, hired labor, which in the long term makes agroforestry more profitable.

To promote the implementation of CAFS and compensate for lower yields that have led to lower adoption, incentives have been proposed for the benefits that this system provides, from an ecological point of view, such as alternative markets, environmental certificates, fair trade, or as a strategy to reduce the problem of climate change. Although cocoa yields are 25% lower in CAFS compared to monoculture, the additional benefits of product diversification make the two systems comparable (Niether et al., 2020).

## Conclusion and perspectives

The works gathered in this review reject the hypothesis that CAFS are always related to biotic and abiotic stresses. Monocultures would be favored in terms of productivity and incidence of diseases compared to CAFS, excluding WBD, since some characteristics such as increased flowering and buds can favor the incidence of this disease in crops with full sun exposure. Therefore, this cultivation condition requires a greater demand for water and nutrients, related to CAFS, representing a concrete threat to productive sustainability, especially considering the projected scenarios of global climate change.

On the other hand, even though some studies report that the incidence of BPR and FPR can be favored in CAFS designs, it is also reported that the correct selection of the model agroforestry and the inclusion of opportune agronomic practices, including biological and cultural control, would mitigate the incidence of these diseases, as well as increase productivity, equaling monocultures. In addition, it is important to emphasize that the CAFS provides ecosystemic services such as the maintenance of pollinators, greater efficiency in the use of water, nutrient demand that may generate a more sustainable production. In the same way, the selection of genotypes with better performance in low light conditions (higher efficiency in the use of light) and better energy balance, can contribute to the mitigation of abiotic stresses normally attributed to CAFS. Therefore, it is necessary to explore the technological and scientific resources that are already available and apply them to production models based on AFS. In addition, it is still necessary to deepen our understanding of the mechanisms triggered by cacao in the face of these complex interactions. Knowing and understanding how these processes work is the key to improving agricultural practices and guaranteeing food security in a future of global climate change.

## Author contributions

JR-M and YJ-S: idea behind this literature review. AC-R, DG-N, and FELC: literature search and the preparation of the draft manuscript. FELC: critical review and exhaustive editing. YJ-S and FELC: revised the final version of the manuscript prior to its submission. All authors contributed to the article and approved the submitted version.

## Funding

The project was financed by the Sistema General de Regalias through the project “Investigación, desarrollo e innovación en cacaos especiales bajo sistemas agroforestales” (BPIN 2013000100255).

## Conflict of interest

The authors declare that the research was conducted in the absence of any commercial or financial relationships that could be construed as a potential conflict of interest.

## Publisher’s note

All claims expressed in this article are solely those of the authors and do not necessarily represent those of their affiliated organizations, or those of the publisher, the editors and the reviewers. Any product that may be evaluated in this article, or claim that may be made by its manufacturer, is not guaranteed or endorsed by the publisher.
